# EEG theta and beta bands as brain oscillations for different knee osteoarthritis phenotypes according to disease severity

**DOI:** 10.1038/s41598-022-04957-x

**Published:** 2022-01-27

**Authors:** Marcel Simis, Marta Imamura, Kevin Pacheco-Barrios, Anna Marduy, Paulo S. de Melo, Augusto J. Mendes, Paulo E. P. Teixeira, Linamara Battistella, Felipe Fregni

**Affiliations:** 1 Hospital das Clinicas HCFMUSP, Faculdade de Medicina, Universidade de São Paulo, São Paulo, Brazil; 2 Neuromodulation Center and Center for Clinical Research Learning, Spaulding Rehabilitation Hospital and Massachusetts General Hospital, Harvard Medical School, 96 13th Street, Charlestown, Boston, MA USA; 3 Universidad San Ignacio de Loyola, Vicerrectorado de Investigación, Unidad de Investigación para la Generación y Síntesis de Evidencias en Salud, Lima, Peru; 4 Psychological Neuroscience Laboratory, CIPsi, School of Psychology, University of Minho, Campus de Gualtar, Braga, Portugal

**Keywords:** Neurology, Chronic pain, Rehabilitation, Biomarkers

## Abstract

This study aims to investigate the multivariate relationship between different sociodemographic, clinical, and neurophysiological variables with resting-state, high-definition, EEG spectral power in subjects with chronic knee osteoarthritis (OA) pain. This was a cross-sectional study. Sociodemographic and clinical data were collected from 66 knee OA subjects. To identify associated factors, we performed independent univariate and multivariate regression models by frequency bands (delta, theta, alpha, beta, low-beta, and high-beta) and by pre-defined regions (frontal, central, and parietal). From adjusted multivariate models, we found that: (1) increased frontocentral high-beta power and reduced central theta activity are positively correlated with pain intensity (β = 0.012, 95% CI 0.004–0.020; and β = − 0.008; 95% CI 0.014 to − 0.003; respectively); (2) delta and alpha oscillations have a direct relationship with higher cortical inhibition; (3) diffuse increased power at low frequencies (delta and theta) are associated with poor cognition, aging, and depressive symptoms; and (4) higher alpha and beta power over sensorimotor areas seem to be a maladaptive compensatory mechanism to poor motor function and severe joint degeneration. Subjects with higher pain intensity and higher OA severity (likely subjects with maladaptive compensatory mechanisms to severe OA) have higher frontocentral beta power and lower theta activity. On the other hand, subjects with less OA severity and less pain have higher theta oscillations power. These associations showed the potential role of brain oscillations as a marker of pain intensity and clinical phenotypes in chronic knee OA patients. Besides, they suggest a potential compensatory mechanism of these two brain oscillators according to OA severity.

## Introduction

Chronic pain associated with osteoarthritis is a leading cause of disability, decreased quality of life, and represents a high economic burden worldwide^1,2^. The perpetuation of pain in knee osteoarthritis (OA) has been associated with maladaptive changes in peripheral and central nervous systems^3,4^. In addition to knee OA, other chronic pain conditions, such as fibromyalgia, orofacial pain, spinal cord injury, and phantom limb pain, also present central nervous system (CNS) alterations like central sensitization of nociceptive pathways, impairment in the descending pain modulation system, and altered emotional-motivational brain systems^1,5–8^.

The use of electroencephalography (EEG)—a noninvasive measure of electrical neuronal activity at different frequencies (delta, theta, alpha, beta, and gamma bands)—has been shown as a promising tool for assessing potential brain function changes in chronic pain patients. Recent EEG studies have shown that chronic pain populations compared to healthy controls have a distinct brain oscillatory signature, mainly an increase in theta and alpha power at rest^9,10^. These results are usually explained by the presence of thalamocortical dysrhythmia (TCD), that is characterized by an increased theta power with decreased dominant alpha activity^8,11–13^. However, these changes are predominately studied in neuropathic pain and not consistently found in musculoskeletal conditions^14–17^.

Besides, fewer studies have explicitly investigated the brain oscillations associated with pain intensity. These studies revealed that objective noxious stimulus intensity was inversely related to alpha and beta oscillations in sensorimotor areas, whereas subjective pain was positively related to oscillations at higher frequencies (high beta and gamma) in frontal areas^17,18^.

Modulating these pain-related brain oscillations can be used as new pain management approach^19–21^. However, most of the current findings are limited by its correlative nature, without considering demographic, clinical, and neurophysiological variables into the analysis as potential confounders or effect modifiers. Therefore, the clinical variables that could influence and predict brain oscillations behavior in chronic pain patients are not completely understood.

Our main hypothesis is that clinical features in patients with chronic pain such as pain intensity, motor function, cognitive-emotional status, quantitative sensory testing, and cortical excitability (indexed by transcranial magnetic stimulation) are associated with specific brain oscillatory patterns at resting EEG. Therefore, this study aims to investigate the multivariate associations of different sociodemographic and clinical variables and EEG brain oscillations of chronic knee OA pain patients. Furthermore, this knowledge may help us understand pain-related brain oscillations, their underlying brain functions, and its potential utility as biomarkers of chronic pain in chronic knee OA patients.

## Results

### Demographics and clinical characteristics

We included 66 patients with knee OA in this study. All participants reported chronic knee OA pain, most of them (96.97%) with bilateral symptoms. The majority of the participants were female, older adults, and overweight or obese. The average pain was moderate (VAS pain of 5.65 [SD = 1.83] from 0 to 10 scale; and Western Ontario and McMaster Universities Osteoarthritis Index (WOMAC) pain of 11.03 [SD = 3.92] from 0 to 20 scale). We found no statistically significant correlation between the WOMAC pain intensity and the K–L grade (p = 0.10). Further baseline clinical and neurophysiological data are provided in Table 1.

**Table 1:** Baseline clinical and sociodemographic characteristics of knee OA study participants.

Variables	Knee OA subjects (N = 66)
**Demographics**	
Age (range)	68.90 ± 9.53 (52–92)
Gender (%)	
Female	61 (92.42)
Male	5 (7.58)
Ethnicity	
White	44 (66.67)
Black	6 (9.09)
Mixed	11 (16.67)
Asian	5 (7.58)
Education level (%)	
Illiterate	1 (1.52)
Elementary	25 (37.88)
High-school	20 (30.30)
Superior	20 (30.30)
Weight (kg)	78.62 ± 13.03
Height (m)	1.56 ± 0.08
BMI	31.88 ± 4.65
**Clinical assessments**	
Bilateral knee OA (%)	64 (96.97)
Time of ongoing pain (months)	97.39 ± 103.89
Total knee arthroplasty (%)	
Right	1 (1.52)
Left	2 (3.03)
Pain—visual analogue scale	
Right	5.96 ± 2.72
Left	5.35 ± 2.76
Average	5.65 ± 1.83
WOMAC total score	51.26 ± 19.01
WOMAC pain	11.03 ± 3.92
WOMAC stiffness	4.64 ± 1.92
WOMAC physical function	35.58 ± 14.34
Kellgren–Lawrence classification	
Right (%)	
1	17 (26.15)
2	15 (23.08)
3	11 (16.92)
4	22 (33.85)
Mean (SD)	2.58 ± 1.21
Left	
1	21 (32.81)
2	15 (23.44)
3	12 (18.75)
4	16 (25)
Mean (SD)	2.36 ± 1.19
Average between right and left	2.50 ± 1.15
Pain Catastrophizing Scale	14.18 ± 10.98
HAM-L Scale	9.36 ± 5.60
Hospital Anxiety and Depression Scale	
Anxiety	5.68 ± 3.93
Depression	4.46 ± 3.56
Montreal Cognitive Assessment	20.67 ± 5.05
Epworth sleepiness scale	11.00 ± 6.19
Quality of life (sf-36)—total score	52.47 ± 20.40
**Quantitative sensory testing**	
Pain pressure threshold	
Upper limb	
Right	5.36 ± 1.96
Left	5.18 ± 2.06
Average	5.27 ± 1.95
Knee	
Right	4.39 ± 2.55
Left	4.39 ± 2.37
Average	4.39 ± 2.42
Conditioned pain modulation	
Right	1.22 ± 1.18
Left	1.01 ± 1.21
Average	1.16 ± 1.04
Average (% of change from baseline)	22.48 ± 22.29
**Transcranial magnetic stimulation**	
Motor threshold	
Right	51.94 ± 11.10
Left	50.30 ± 10.58
Average	51.12 ± 9.86
Motor evoked potential	
Right	1.64 ± 1.08
Left	1.72 ± 1.44
Average	1.68 ± 1.02
Cortico-silent period	
Right	93.20 ± 35.51
Left	82.32 ± 32.54
Average	87.76 ± 31.64
Short intracortical inhibition	
Right	0.46 ± 0.32
Left	0.47 ± 0.31
Average	0.47 ± 0.26
Intracortical facilitation	
Right	1.59 ± 0.71
Left	1.63 ± 0.82
Average	1.61 ± 0.60

### Delta band oscillations models

Cognitive function (as indexed by the Montreal Cognitive Assessment [MoCA] scale) has been negatively associated with delta oscillation activity in the frontal (β = − 0.008, 95% CI − 0.013 to − 0.003; p = 0.002), central (β = − 0.009, 95% CI − 0.014 to − 0.004; p = 0.001), and parietal (β = − 0.006, 95% CI − 0.012 to − 0.001; p = 0.013) regions. Short interval intracortical inhibition (SICI) was negatively associated with delta band activity in the frontal (β = − 0.137, 95% CI − 0.232 to − 0.042; p = 0.005) and central (β = − 0.100, 95% CI − 0.194 to − 0.006; p = 0.037) areas, indicating a direct relationship between delta oscillation and intracortical inhibition. Furthermore, a significant, positive relationship between age and delta activity can be observed in the frontal (β = 0.004, 95% CI 0.002–0.007; p = 0.001) and parietal (β: 0.003, 95% CI 0.0006–0.006; p = 0.015) regions, suggesting an increase in delta activity with aging. Moreover, depression (as indexed by the Hamilton Depression Rating Scale [HAM-L]) was positively associated with delta activity in the frontal (β = 0.007, 95% CI 0.002–0.011; p = 0.001) and parietal (β = 0.007, 95% CI 0.002–0.012; p = 0.004) areas. Finally, social function (as indexed by the SF-36 questionnaire) was also identified as associated with delta band activity with a positive relationship, in all regions (frontal: β = 0.002, central: β = 0.001, and parietal: β = 0.007). Time of ongoing pain was the main confounder identified in delta band models (Table 2).

**Table 2:** Baseline delta band multivariate analyses according to region of interest (ROI).

Variables	Beta-coefficient	95% CI	p-value	R^2^
**Frontal region**				0.440
MoCA score	− 0.008	− 0.013 to − 0.003	0.002	
SF-36 social function subscale	0.002	0.001 to 0.003	< 0.001	
SICI average	− 0.137	− 0.232 to − 0.042	0.005	
HAM-L depression scale	0.007	0.002 to 0.011	0.004	
Age	0.004	0.002 to 0.007	0.001	
Time of ongoing pain	− 0.0001	− 0.0003 to 0.0001	0.312	
**Central region**				0.291
MoCA score	− 0.009	− 0.014 to − 0.004	0.001	
SF-36 social function subscale	0.001	0.0004 to 0.002	0.005	
SICI average	− 0.100	− 0.194 to − 0.006	0.037	
Time of ongoing pain	− 0.0001	− 0.0003 to 0.0001	0.339	
Age	0.002	− 0.0005 to 0.004	0.114	
**Parietal region**				0.344
MoCA score	− 0.006	− 0.012 to − 0.001	0.013	
HAM-L depression scale	0.007	0.002 to 0.012	0.004	
SF-36 social function subscale	0.002	0.0009 to 0.003	< 0.001	
Age	0.003	0.0006 to 0.006	0.015	
Time of ongoing pain	− 0.0002	− 0.0004 to 0.00004	0.109	

### Theta band oscillations models

**Figure 1:**
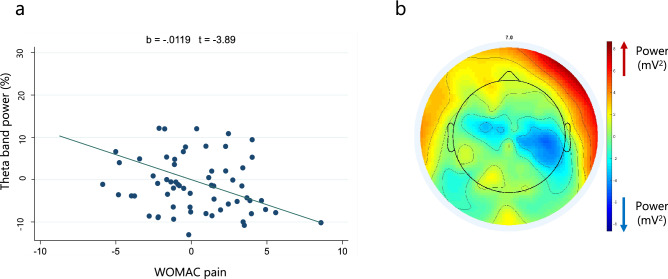
(**a**) Theta band power and pain intensity adjusted correlation (from multivariate model of central region). (**b**) Topographical plot of spectral power from representative subjects (n = 15) with high pain intensity (higher than ten in WOMAC pain scale).

In relation to theta oscillations, pain intensity (as indexed by the WOMAC pain scale) is conveyed as having a statistically significant, negative association with theta activity in the frontal (β = − 0.012; 95% CI − 0.018 to − 0.006; p < 0.001), central (β = − 0.008; 95% CI 0.014 to − 0.003; p = 0.001), and parietal (β = − 0.008; 95% CI − 0.013 to − 0.002; p = 0.006) areas. This negative correlation is presented in the Fig. 1a and corroborated by the topographical map from representative patients with high pain intensity (Fig. 1b). In contrast, positive associations between motor function (indexed by timed up and go test) and theta activity was seen in the frontal (β = 0.003; 95% CI 0.001–0.005; p = 0.003), central (β = 0.005, 95% CI 0.002–0.007; p < 0.001), and parietal (β = 0.005, 95% CI 0.002–0.008; p < 0.001) regions. Besides, we found female participants have statistically significant higher theta band power in all three areas (frontal: β = 0.083; central β = 0.104; and parietal: β = 0.114). Moreover, OA severity was shown to be negatively correlated to theta oscillations in the central (β = − 0.023; 95% CI − 0.042 to − 0.004; p = 0.019) and parietal (β = − 0.025; 95% CI − 0.046 to − 0.004; p = 0.019) regions. Depression was only significantly correlated with theta bands in the frontal (β = 0.008; 95% CI 0.0004–0.017; p = 0.039) region, conveying a positive correlation (Table 3).

**Table 3:** Baseline theta band multivariate analyses according to ROI.

Variables	Beta-coefficient	95% CI	p-value	R^2^
**Frontal region**				0.330
WOMAC pain scale	− 0.012	− 0.018 to − 0.006	< 0.001	
Timed up and go score	0.003	0.001 to 0.005	0.003	
HAD-depression scale	0.008	0.0004 to 0.017	0.039	
Gender (female)	0.083	0.006 to 0.159	0.034	
HAD-Anxiety scale	− 0.004	− 0.011 to 0.003	0.238	
**Central region**				0.374
WOMAC pain scale	− 0.008	− 0.014 to − 0.003	0.001	
Timed up and go score	0.005	0.002 to 0.007	< 0.001	
Gender (female)	0.104	0.031 to 0.177	0.006	
Kellgren–Lawrence scale	− 0.023	− 0.042 to − 0.004	0.019	
Age	0.0007	− 0.002 to 0.003	0.556	
**Parietal region**				0.353
Timed up and go total	0.005	0.002 to 0.008	< 0.001	
WOMAC pain scale	− 0.008	− 0.013 to − 0.002	0.006	
Gender (female)	0.114	0.034 to 0.193	0.006	
Kellgren–Lawrence scale	− 0.025	− 0.046 to − 0.004	0.019	
Age	0.001	− 0.001 to 0.004	0.429	

### Alpha band oscillations models

We found that equivalent set of variables were associated with alpha band oscillations in the frontal, central, and parietal regions. We found a statistically significant positive relationship with motor evoked potentials (MEP) (b = 0.040, b = 0.032, and b = 0.045 in frontal, central, and parietal regions, respectively); with cortical silent period (CSP) (b = 0.001, b = 0.001, b = 0.001, respectively), indicating a direct relationship between alpha oscillation and cortical inhibition; and with WOMAC stiffness score (b = 0.020, b = 0.019, b = 0.022, respectively). Additionally, an association with gender was found, namely female participants have lower alpha band power in all areas (b = − 0.123, b = − 0.137, b = − 0.164, respectively). Depression scale was found as the main confounder of alpha band oscillations (Table 4).

**Table 4:** Baseline alpha band multivariate analyses according to ROI.

Variables	Beta-coefficient	95% CI	p-value	R^2^
**Frontal region**				0.280
MEP average	0.040	0.010 to 0.071	0.011	
CSP average	0.001	0.0002 to 0.002	0.014	
Gender (female)	− 0.123	− 0.241 to − 0.005	0.042	
WOMAC stiffness score	0.020	0.003 to 0.037	0.021	
HAM-L depression scale	− 0.005	− 0.011 to 0.001	0.122	
**Central region**				0.271
Gender (female)	− 0.137	− 0.251 to − 0.023	0.019	
MEP average	0.032	0.002 to 0061	0.036	
CSP average	0.001	0.0002 to 0.002	0.014	
WOMAC stiffness score	0.019	0.002 to 0.035	0.028	
HAM-L depression scale	− 0.004	− 0.010 to 0.001	0.153	
**Parietal region**				0.247
MEP average	0.045	0.006 to 0.083	0.023	
Gender (female)	− 0.164	− 0.312 to − 0.016	0.030	
CSP average	0.001	0.0001 to 0.002	0.032	
WOMAC stiffness score	0.022	0.0003 to 0.043	0.047	
HAM-L depression scale	− 0.006	− 0.013 to 0.002	0.138	

### Beta band oscillations models

In the beta band we found distinct set of associated variables in each ROI. Regarding the frontal region, we found a positive association of WOMAC pain (b = 0.013, 95% CI 0.003–0.024, p = 0.010), upper limb PPT (b = 0.031, 95% CI 0.012–0.050, p = 0.002), and OA severity (K–L scale) (b = 0.033, 95% CI 0.004–0.062, p = 0.027). The bilateral affectation status (i.e., whether the subject have bilateral symptoms or not) and depression scale were maintained in the models as confounders. In the central region, similar positive relationship was found regarding OA severity (Kellgren–Lawrence Scale) (b = 0.044, 95% CI 0.012–0.075, p = 0.007). Additionally, a negative association was found with Timed Up and Go score (b = − 0.004, 95% CI − 0.008 to − 0.0003, p = 0.037). CSP and WOMAC stiffness score were kept in the model as confounders. At last, in the parietal region, a similar positive association was found with OA severity (K–L scale) (b = 0.064, 95 CI 0.029–0.099, p = 0.001). We also found a positive relationship with Berg Balance Scale (b = 0.006, 95% CI 0.002–0.011, p = 0.006) and a negative one with CSP (b = − 0.001, 95% CI − 0.002 to − 8.520, p = 0.048). The bilateral affectation was the main confounder in the model (Table 5).

**Table 5:** Baseline beta band multivariate analyses according to ROI.

Variables	Beta-coefficient	95% CI	p-value	R^2^
**Frontal region**				0.240
WOMAC pain scale	0.013	0.003 to 0.024	0.010	
Upper limb PPT	0.031	0.012 to 0.050	0.002	
Kellgren–Lawrence scale	0.033	0.004 to 0.062	0.027	
Bilateral affectation	0.024	− 0.026 to 0.074	0.343	
HAD-depression scale	− 0.008	− 0.020 to 0.003	0.133	
**Central region**				0.161
Kellgren–Lawrence scale	0.044	0.012 to 0.075	0.007	
Timed up and go score	− 0.004	− 0.008 to − 0.0003	0.037	
CSP average	− 0.001	− 0.002 to 0.00005	0.063	
WOMAC stiffness scale	0.006	− 0.010 to 0.022	0.471	
**Parietal region**				0.259
CSP average	− 0.001	− 0.002 to − 8.520	0.048	
Kellgren–Lawrence scale	0.064	0.029 to 0.099	0.001	
Berg balance scale	0.006	0.002 to 0.011	0.006	
Bilateral affectation	0.027	− 0.025 to 0.080	0.299	

### Sensitivity analysis by beta sub-bands

#### Low-beta band oscillations models

In this sub-band, we confirmed the positive relationship with WOMAC pain in the frontal (b = 0.006, 95% CI 0.002–0.010, p = 0.006) and central regions (b = 0.005, 95% CI 0.0007–0.010, p = 0.023). Also, the direct association with OA severity in frontal (b = 0.013, 95% CI 0.0007–0.025, p = 0.038), central (b = 0.014, 95% CI 0.0007–0.028, p = 0.039), and parietal regions (b = 0.026, 95% CI 0.009–0.044, p = 0.003). Similarly, to the beta band model, we found a positive correlation with PPT, only present in the frontal region but neither in central nor parietal areas; and a direct relationship with Berg balance score, but only in the parietal region. Moreover, we found a differential association in this sub-band, a positive correlation with SF-36 emotion subscale for frontal and central, but not for parietal areas. In all the low-beta band models, the bilateral affectation status was the only identified confounder (Table 6).

**Table 6:** Baseline low beta band multivariate analyses according to ROI.

Variables	Beta-coefficient	95% CI	p-value	R^2^
**Frontal region**				0.271
WOMAC pain scale	0.006	0.002 to 0.010	0.006	
Upper limb PPT	0.011	0.003 to 0.012	0.005	
SF-36 emotion subscale	0.0004	0.00008 to 0.0007	0.014	
Kellgren–Lawrence scale	0.013	0.0007 to 0.025	0.038	
Bilateral affectation	0.008	− 0.012 to 0.029	0.417	
**Central region**				0.284
SF-36 emotion subscale	0.0006	0.0002 to 0.0009	0.001	
Kellgren–Lawrence scale	0.014	0.0007 to 0.028	0.039	
Upper limb PPT	0.007	− 0.0009 to 0.016	0.079	
WOMAC pain scale	0.005	0.0007 to 0.010	0.023	
Bilateral affectation	0.017	− 0.006 to 0.040	0.138	
**Parietal region**				0.186
Berg balance scale	0.003	0.0007 to 0.005	0.001	
Kellgren–Lawrence scale	0.026	0.009 to 0.044	0.003	
Bilateral affectation	0.018	− 0.008 to 0.044	0.169	

#### High-beta band oscillations models

**Figure 2:**
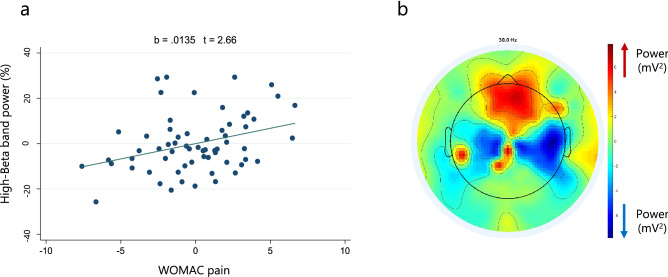
(**a**) High-beta band power and pain intensity adjusted correlation (from multivariate model of frontal region). (**b**) Topographical plot of spectral power from representative subjects (n = 15) with high pain intensity (higher than 10 in WOMAC pain scale).

Regarding this sub-band, equally to beta and low-beta band models, we confirmed the robust positive association with WOMAC pain in the frontal region (b = 0.012, 95% CI 0.004–0.020, p = 0.004). This correlation is presented in the Fig. 2a and corroborated by the topographical map from representative patients with high pain intensity (Fig. 2b). Likewise, we verified the direct relationship with OA severity in frontal (b = 0.039, 95% CI 0.005–0.029, p < 0.001), central (b = 0.027, 95% CI − 0.008 to 0.047, p = 0.007), and parietal areas (b = 0.035, 95% CI 0.014–0.055, p = 0.001), as well as with upper limb PPT in the frontal region (b = 0.017, 95% 0.005–0.029, p = 0.005). Additionally, we confirmed the negative association with Timed Up and Go score (b = − 0.002, 95% CI − 0.005 to − 0.00006, p = 0.045) and 10-m walking test score (b = − 0.004, 95% CI − 0.007 to − 0.0003, p = 0.034) in the central and parietal regions, respectively. Moreover, we corroborated the negative association with CSP in the central (b = − 0.0008, 95% CI − 0.001 to − 0.0002, p = 0.010) and parietal (b = − 0.0009, 95% CI − 0.001 to − 0.0002, p = 0.010) areas. Finally, we found a differential association in this sub-band, a positive correlation with SF-36 physical function subscale for frontal and parietal, but not for central regions. The bilateral affectation, Hospital Anxiety and Depression (HAD)-Depression scale and WOMAC stiffness were the identified confounders in those models (Table 7).

**Table 7:** Baseline high beta band multivariate analyses according to ROI.

Variables	Beta-coefficient	95% CI	p-value	R^2^
**Frontal region**				0.320
WOMAC pain scale	0.012	0.004 to 0.020	0.004	
Upper limb PPT	0.017	0.005 to 0.029	0.005	
Kellgren–Lawrence scale	0.039	0.018 to 0.060	< 0.001	
SF-36 Physical function scale	0.002	0.0008 to 0.003	0.002	
Bilateral affectation	0.012	− 0.019 to 0.044	0.435	
HAD-depression scale	− 0.001	− 0.008 to 0.006	0.696	
**Central region**				0.196
CSP average	− 0.0008	− 0.001 to − 0.0002	0.010	
Kellgren–Lawrence scale	0.027	− 0.008 to 0.047	0.007	
Timed Up and Go score	− 0.002	− 0.005 to − 0.00006	0.045	
WOMAC stiffness scale	0.005	− 0.005 to 0.015	0.343	
**Parietal region**				0.271
CSP average	− 0.0009	− 0.001 to − 0.0002	0.010	
SF-36 physical function scale	0.0006	0.00005 to 0.001	0.034	
Kellgren–Lawrence scale	0.035	0.014 to 0.055	0.001	
10-m walking test score	− 0.004	− 0.007 to − 0.0003	0.034	

The summary of all significant predictors of brain oscillations in chronic knee osteoarthritis pain is presented in Table 8.

**Table 8:** Findings summary from multivariate models by brain oscillation and ROI.

Relative power	Frontal	Central	Parietal
**↑ **Delta	↑ SF-36 social functioning	↑ SF-36 social functioning	↑ SF-36 social functioning
	↑ Depression (Hamilton)		↑ Depression (Hamilton)
	↑ Age		↑ Age
	↓ MOCA test	↓ MOCA test	↓ MOCA test
	↓ SICI (higher inhibition)	↓ SICI (higher inhibition)	
**↑ **Theta	↑ Timed up and go test	↑ Timed up and go test	↑ Timed up and go test
	↑ in women	↑ in women	↑ in women
	↑ Depression (HAD)		
	↓ Pain (WOMAC)	↓ Pain (WOMAC)	↓ Pain (WOMAC)
		↓ KL severity	↓ KL severity
**↑ **Alpha	↑ MEP	↑ MEP	↑ MEP
	↑ Cortical silent period	↑ Cortical silent period	↑ Cortical silent period
	↑ WOMAC stiffness	↑ WOMAC stiffness	↑ WOMAC stiffness
	↓ in women	↓ in women	↓ in women
**↑ **Beta	↑ WOMAC pain	↑ KL severity	↑ KL severity
	↑ Pain threshold (hand)		↑ Balance test (EEB)
	↑ KL severity		
		↓ Timed up and go test	↓ Cortical silent period
**↑ **Low beta	↑ SF-36 emotional functioning	↑ SF-36 emotional functioning	↑ Balance test (EEB)
	↑ KL severity	↑ KL severity	↑ KL severity
	↑ Pain (WOMAC)	↑ Pain (WOMAC)	
	↑ Pain threshold (Hand)		
**↑ **High beta	↑ KL severity	↑ KL severity	↑ KL severity
	↑ SF-36 emotional functioning		↑ SF-36 physical functioning
	↑ Pain threshold (Hand)		
	↑ Pain (WOMAC)		
		↓ Cortical silent period	↓ Cortical silent period
		↓ Timed up and go test	↓ Walking test speed (10 MWT)

## Discussion

### Main findings

This study aimed to explore the association of different sociodemographic, clinical, and neurophysiological variables and the resting-state EEG spectral power in subjects with chronic pain due to knee OA. Based on previous systematic review^10^, this is one of the largest studies exploring the brain oscillations correlates in chronic knee OA pain using a multivariate approach, looking to understand better pain-related oscillatory activity, their underlying brain processes, and its potential utility as biomarkers of chronic pain. Our main findings showed important relationships between clinical and demographic variables and EEG power: (1) multivariate analyses showed that higher pain intensity and higher OA severity (indexed by K–L scale) is associated with higher frontocentral beta and high-beta power and a reduction of diffuse theta activity; (2) delta and alpha oscillations have a direct relationship with higher cortical inhibition (SICI and CSP, respectively); (3) increased power at low frequencies (delta and theta) are associated with poor cognition, aging, and depressive symptoms; (4) higher alpha and beta power seems to be a maladaptive compensatory mechanism to poor motor function and severe joint degeneration; and (5) gender seems to be an important biological variable, acting as confounder in pain-related brain oscillations assessment.

### Brain oscillations and pain intensity

#### Theta oscillations and pain

Increased theta band activity is shown to be positively related to pain in different chronic pain conditions such as fibromyalgia, spinal cord injury (SCI), and other forms of neuropathic pain^22,23^. This relationship has been justified by the theoretical framework of thalamocortical dysrhythmia (TCD), which is thought to originate from abnormal oscillatory activity and interference to increase pain^24,25^. Interestingly, our findings convey a negative correlation between theta band oscillation and pain in individuals with knee OA, similar to a previous study in hip OA^26^. It is worth mentioning that OA consists of a mixed phenotype of pain mechanisms, completely distinct from the mechanisms observed in fibromyalgia and in neuropathic pain^27,28^.

One interesting aspect of theta rhythm is that it seems to be correlated with emotional control. In fact, a group of investigators, in a previous study with 30 healthy subjects, showed that subjects with high theta power especially in midline structures had low anxiety scores^29^. Thus, interestingly our results showing that high theta is associated with less pain but at the same time with less OA severity and also being more frequent in women, likely points out to a potential affective control of pain. Studies have also shown that increased theta is associated with higher metabolic activity in the anterior cingulate cortex (ACC). Therefore, we can hypothesize that in musculoskeletal disorders, theta may be a modulator of affective networks associated with pain control and does not support the TCD framework.

#### Beta oscillations and pain

Our results also show that higher beta power, in frontocentral areas are associated with higher self-reported pain intensity during functional activities as measured by the WOMAC pain subscale. It is also important to underscore that beta increase was also correlated with a greater OA severity, although WOMAC pain and K–L grade were not correlated in our sample In this context, increased beta seems to be related to a compensatory mechanism of greater neuronal injury and representing a subgroup of patients with less adaptative response and potentially higher central sensitization in response to the chronic joint degeneration. Such finding can be seen also in other examples of neural injury such as in stroke^30,31^ and spinal cord injury^8,32,33^. In fact, beta oscillations seem to be related to increased local metabolic activity^34,35^, thus likely in the case of OA generating additional electric activity to compensate for the OA indirect neural lesion. When looking at studies on musculoskeletal pain conditions, our results agree with previous reports on chronic hip OA^17^ and low back pain^36^ where higher frequency brain oscillations in frontal areas are associated with higher pain intensity. A potential explanation relies on the evidence that supports the link between the presence of beta oscillations and cortical dysfunction in motor impairment conditions^37,38^, as well as its association with cortex activation during motor tasks^39,40^.

### Cortical inhibition and brain oscillations

Studies have found strong correlations between cortical silent period (CSP) and alpha band oscillations, indicating its potential as an indicator of inhibitory processes^41^. Moreover, our finding, of this relationship in other studied thus indicate alpha oscillation's inhibitory race between brain regions, not only within them, implying a state of lowered excitability and heightened inhibition in patients with high alpha band oscillations^42^. Our results also found a positive correlation between motor evoked potential (MEP) and alpha bands. This finding can be associated with the positive correlation between alpha band and CSP given MEP modulates cortical-spinal and the cortical-spinal pathway requires cortical inhibition. Moreover, studies evaluating markers of cortical excitability have found that markers such as intracortical facilitation and short-interval intracortical inhibition affect changes in MEP, suggesting that MEP is an unreliable biomarker for cortical excitability^43^.

Regarding delta bands, we found a negative relationship with short intracortical inhibition (SICI), that means that higher delta is associated with higher intracortical inhibition in this sample. Delta band activity is thought of influencing cortical facilitation in different brain pathways^44^. A negative relationship between SICI and delta waves observed in this study convey that, individuals with high delta activity have more inhibitory functions in the frontal and central cortical regions. This finding is consistent with studies that report delta is shown to be significantly involved in cognitive processes throughout the brain. Studies have found that inhibition of specific pathways in the cortex contribute to increase focus and attention required to perform certain cognitive tasks^44^. Therefore, our findings suggest that delta might be a good marker for cortical inhibitory activity.

### Emotional-cognitive systems and low-frequency bands

A consistent finding within the signature of EEG low-frequency bands is their association with depression^45^. Our model depicts theta activity directly associated with depression scores in the frontal and parietal regions, supporting the increase in neuro physiologic connectivity depicted by delta, theta, and beta bands in other studies^46^. It is thought that high theta activity in individuals with major depressive disorder (MDD) functions as a compensatory mechanism in response to cortical deficits cause by MDD. In return, a negative relationship between delta activity is observed in individuals with MDD due to this cortical deficit^47^. Although a positive relationship between delta oscillations and depression in our results may be seem as a contradictory finding at the first glance, we see the opposite. It does confirm these previous studies. Given that our individuals in our sample do not have MDD, and only exhibit sub-clinical symptoms of depression, subjects with increased depression scores are those with active delta likely as a compensatory marker compared to individual with no depression symptoms. It is the issue of correlational tests. In this case we believe here that subjects with depression elicit higher delta as a compensatory mechanism and not the opposite. For that reason, we do not expect the classic conclusions of MDD brain oscillation activity. Moreover, delta waves have been shown to affect motivational and reward areas of the cortex, suggesting its influence on mood brain functions^48^.

Moreover, an inverse relationship was found between cognition and delta oscillations. This finding is consistent with studies that convey associations between increased delta and dementia in individuals with Parkinson Disease (PD)^49^. Although this result may seem conflicting with the relationship of delta and SICI, cognitive decline associated with increased delta activity may be related to different causal pathways than those that relate delta inhibitory function with attention. This hypothesis can be supported by the positive relationship between delta waves and age also found in our study. Cognitive decline is associated with aging and delta activity is increased in both or these processes^50^. Thus, age and cognition could be possible predictors of delta activity in patients w/one in the chronic pain condition.

### Brain oscillations, poor motor function, and severe OA

Consistent with the association between pain and theta bands, a positive correlation between theta bands and motor function was observed in our models. Increased theta band activity is required to trigger fine initiation of lower-limb movement in individuals with PD^51^. In the context of OA, more movement is associated with less pain, therefore individuals with high theta activity display higher motor function and motor control, which relates to the inverse relationship between theta activity and pain; individuals with better motor function have less pain, observed in individuals with increased theta oscillations, according to this study’s results^45^.

The theta band signature in OA patients is further strengthened by the negative association found with disease severity. Disease severity is reportedly colinear to pain intensity, thus, the inverse relationships observed between theta, pain, and disease severity suggest theta band activity as a potential biomarker for individuals with OA pain.

Besides, our results also showed that higher beta power in centroparietal areas associated with poor balance and motor function. This finding concurs with previous research that has found that high-beta EEG oscillations power can predict motor recovery in spinal cord injury patients^33^. Thus, we hypothesize that pain processing in knee OA requires a balanced and harmonized cortex activation of which the high beta frequency over frontal areas can potentially serve as a signature of a compensatory pattern of high frequencies oscillatory activity in response to a dysfunctional cortical-subcortical pain regulation caused by chronic inflammation and movement impairment associated with the OA condition.

An unexpected finding in this study was the positive association between alpha bands and stiffness (as indexed by the WOMAC Stiffness scale). Individuals with osteoarthritis commonly report stiffness and rigidity in the affected joints in the mornings or after long periods of being still^52^. Considering stiffness has some degree of collinearity with disease severity in OA and the role of alpha band oscillations in cortical inhibition, this association might indicate a compensatory inhibitory mechanism in which peripheral signals of disease severity, such as cartilage destruction and osteophyte formation, might trigger cortical inhibition causing an increase in alpha oscillations, and thus increasing stiffness and restricting movement.

### Gender differences and brain oscillations

Reported gender differences regarding high alpha and theta relative power was found in our study. Not many studies have accounted for gender when evaluating alpha wave oscillation differences in individuals with chronic pain. However, given that different gender exhibit different pain mechanisms in the context of chronic pain, it is reasonable to hypothesize that those same differences might be present in EEG band oscillation changes, particularly those related to pain, such as theta bands^53^. Moreover, a study evaluating resting-brain differences in male and female individuals have suggested differences in cortical excitability in different genders, thus, given the relationship between CSP and alpha band, it is likely that the association between alpha band activity and females in this study supports this finding^54^. Further studies are needed to explore the differences between genders on pain-related brain oscillations, but also a carefully inclusion of gender as mandatory covariate in classic EEG analysis plan.

### Future perspectives

One of our main results was the suggestion of two potential EEG-based pain phenotypes in chronic pain due to knee OA. Patients with higher pain intensity and OA severity (K–L grade) have higher beta band power in the frontocentral regions. On the other hand, patients with low pain intensity and less OA severity have higher diffuse theta band power. As reported by previous studies^55^, chronic pain appears to be associated with abnormal oscillations at theta and beta frequencies. One potential use is the validation of a brain-based biomarker of pain severity and central sensitization^56–58^, which is highly needed considering the subjective metrics we are using to assess this condition in the clinic. Another potential application is using these EEG signatures to guide and stratify pain treatments among patients with chronic pain due to knee OA^59,60^. Due to the potential difference in central maladaptive mechanisms between these two subgroups of patients, likely more neuroplasticity-oriented treatments (such as noninvasive brain stimulation) could have better clinical effects. Finally, these main EEG findings can be used as targets for special neuromodulatory techniques such as transcranial alternating current stimulation (tACS) and neurofeedback^61^. These techniques can be used to revert the high frontocentral beta oscillations associated with higher pain or to induce higher theta band power associated with less pain intensity. However, the potential applicability of these results warrants future confirmatory explorations before its clinical use in chronic pain.

### Limitations

The main limitation of our study is its exploratory nature; thus, no adjustment for multiple comparisons was performed. Future confirmatory research is needed to test and validated our findings as markers of different pain phenotypes in chronic OA pain. Furthermore, the lack of control group could be considered a limitation; however, since our main objective was to describe the associations of EEG and chronic OA clinical variables, the use of healthy controls or other rheumatological disease would be inappropriate. Finally, as chronic pain is affected by a wide range of factors, as medications and comorbidities that could affect also the neurophysiological and pain-related measurements (such as mental disorders, diabetes, and peripheral neuropathies), it is a challenge to control all of them and some influences in the pain perception and EEG findings could be overlooked in our study.

## Conclusions

In summary, our study could identify clear associations of demographic, clinical, and neurophysiological variables, and resting-state EEG spectral power in patients with chronic knee osteoarthritis pain. These associations showed the potential role of brain oscillations as a marker of pain intensity and clinical phenotypes in chronic pain patients. Subjects with higher pain intensity (likely subjects with maladaptive compensatory mechanisms to poor motor function and severe joint degeneration) have higher frontocentral beta power and lower central theta activity. Also, it is important to note that brain oscillation at low frequencies are significantly affected by cognitive and emotional factors, suggesting its potential use for phenotyping clinical profiles of chronic knee osteoarthritis patients. However, our study has some limitations regarding our methodology and the generalizability of our results. Finally, the suggested cortical inhibitory nature (indexed by SICI and CSP) of frontal delta and alpha oscillations underscore the opportunity of modulating pain-related oscillations as new pain management approach. More research is needed with broader and more general samples to bring more consistency for the role of EEG as pain biomarker.

## Methods

### Study design

We performed a cross sectional analysis of patients with knee OA from an ongoing, prospective cohort study titled “Deficit of Inhibition as a Marker of Neuroplasticity (DEFINE study) in rehabilitation” (protocol paper under review). The DEFINE protocol and this study were approved by the Research and Ethical Comitee of Hospital das Clínicas da Faculdade de Medicina da Universidade de São Paulo (HC FMUSP) **(**Registration number: 86832518.7.0000.0068). All the proceedings and methods of this study are in accordance with Brazilian research ethics regulations and the Declaration of Helsinki.

### Participants

#### Inclusion criteria

Adults (over 18 years old), male and female, clinical and radiological diagnosis (magnetic resonance imaging or computerized tomography; or bilateral knee radiography) of knee OA, clinical stability verified by medical evaluation, informed consent form signed by the subject, and meet the eligibility criteria for the Instituto de Medicina Fisica e Reabilitacao (IMREA) rehabilitation program (protocol paper under review).

#### Exclusion criteria

Subjects were excluded if they were pregnant, have active OA with clinical manifestations in joints other than the knee, or if they had any other clinical or social conditions that interfere with the patient’s participation in the rehabilitation program^62^. We did not exclude patients with adequate functionality but with specific comorbidities (such as hypertension or diabetes).

### Study procedures

Patients admitted to the IMREA’s conventional rehabilitation program with knee OA were invited to participate in the study and included after signing the informed consent form. During one visit, a qualified researcher performed a series of clinical and neurophysiological assessments. Instruments were selected to enable a global assessment of patients. Evaluations were carried out by the same examiner. The evaluators were trained to standardize the assessments.

### Demographic and clinical assessments

Information regarding the participants’ age, gender, time of ongoing pain, height, weight, and body mass index were collected from a standardized medical interview. We assessed pain intensity using visual analog scale and the Western Ontario and McMaster Universities Osteoarthritis Index (WOMAC) pain scale. The OA severity was assessed by Kellgren Lawrence radiographic classification. Besides, in order to characterize the study’s sample, we performed a multidimensional assessment including quality of life (SF-36), functional status (WOMAC), motor (Timed up and Go [TUG], 6-min and 10-m walk test), cognitive (MOCA test), sleep (Epworth sleepiness scale), and emotional (Hamilton depression scale, Hospital anxiety and depression scale) functions using standardized scales. A summary of all assessments can be seen in the Supplementary material S1.

### Static and dynamic quantitative sensory testing (QST)

#### Pressure pain threshold (PPT)

We used an algometer to define the minimum amount of pressure that triggers pain in pre-established regions (thenar region, and region located one inch above the knee)^63^. We performed three algometry measurements (15-s intervals) and calculated the average.

#### Conditioned pain modulation (CPM)

We used the CPM response as measurement of changes in pain processing. This test assessed, through intense heterotopic stimulation, the response of the descending pain inhibitory system^64,65^. Producing a “pain inhibits pain” phenomenon^66^. Based on previous studies^67,68^, subjects immersed one of their hands into a recipient containing cold water (10–12 °C) for one minute. After 30 s of immersion, the Visual Analogue Scale (VAS) was presented to patients to indicate their pain level, referring to the submerged hand. Subsequently, three algometric measures (PPTs) were taken (spaced between 15 s) for the contralateral hand. After an interval of approximately 10 min (time for hand to return to normal body temperature), the other hand was immersed in the recipient, and follow the previously stated protocol^68^. CPM response was calculated as the difference between the average PPTs minus the average PPTs during the conditioned stimulus.

### Transcranial magnetic stimulation (TMS)

The Magstim Rapid^®^ stimulator (The Magstim Company Limited, UK). We placed a 70 mm coil in figure-of-eight at 45° of the scalp, to send a perpendicular pulse over the right and left motor cortex (for all assessments), the coil stability and direction was managed by the assessor without neuronavigation. The muscular response to the stimulus was recorded using surface electromyography (EMG) with Ag/AgCl electrodes positioned on first dorsal interosseous (FDI) muscle of the hand and the grounding electrode positioned on the wrist^69^.

We performed a bilateral upper limb assessment. We used anatomical references for motor cortex localization. Initially, we identified the vertex (intersection between the nasion-inion lines and zygomatic arches); then, a mark was made 5 cm from the vertex towards the ear tragus in the coronal plane. The hotspot was determined as the location with the highest and most stable motor evoked potential (MEP) amplitudes over the FDI. The resting motor threshold (rMT) was defined as the minimum intensity necessary for a single TMS pulse on the hot spot to generate an MEP, with at least 50 μV peak to peak amplitude, in 50% of attempts^70^. We performed the following measures: MEP (intensity at 120% of rMT, we calculated the peak-to-peak amplitude), cortical silent period (CSP), which represents the temporary suppression of electromyographic activity during a sustained voluntary contraction. Moreover, we performed paired-pulse protocols of intracortical inhibition (SICI), which was assessed by interstimulus intervals of 2 ms; and intracortical facilitation (ICF) assessed by 10 ms interim stimulus intervals^70^. Ten randomized stimuli were applied at each interval and the average were calculated.

For the measurement of neurophysiological markers through TMS, we pooled the rMT, CSP, SICI, ICF, and MEP results from each hemisphere to obtain a bi-hemispheric average. This approach can be justified due to the bi-hemispheric nature of pain perception^71^; besides, most of our sample includes patients with bilateral knee OA. We then analyzed the relationship between the bi-hemispheric average of these neuro markers with possible associated variables to their behavior (markers magnitude and direction), including clinical and sociodemographic subject characteristics. TMS data was recorded and stored in a computer for off-line analysis.

### Resting-state electroencephalography (EEG)

#### EEG acquisition

We recorded the EEG following a standardized approach^72^. Recordings were performed in a quiet room. Patients were asked to sit in a comfortable position, have their sight directed naturally below the horizon line, not to move or talk, and relax as much as possible. The investigator made sure they did not fall asleep by observing the patient and verbally calling his attention if drowsiness was noticed. Resting-state EEG was recorded for 5 min with eyes closed using a 128-channel EGI system (Electrical Geodesics, Inc) (EGI, Eugene, USA). The EEG was recorded with a band-pass filter of 0.3–200 Hz and digitized at the sampling rate of 250 Hz.

#### Resting-state spectral power analysis

The data were exported for offline analysis with EEGLab^73^ and MATLAB (MATLAB R2012a, The MathWorks Inc. Natick, MA, 2000). EEG was re-referenced to the average, we used finite impulse response filters, one high-pass filter of 1 Hz and a low-pass filter of 40 Hz, followed by manual artifact detection and rejection by a blinded assessor to exclude the existence of any signal of drowsiness (attenuation of the alpha rhythm), epileptiform or any abnormal discharges prior to admission into full study (no epileptiform or abnormal discharges were found). This analysis was followed by a manual artifact detection and rejection and Independent Component Analysis (ICA); finally, we removed the ICs associated to artifacts and reconstructed the signal^74^. The artifact-free data was processed using *pop_spectopo* EEGLab function with Fast Fourier Transformation with 5 s windows with 50% overlap. Absolute power (μV2) and relative power (power in a specific frequency range/total power from 1 to 40 Hz) were calculated for the following frequency bands: delta (1–4 Hz), theta (4–8 Hz), alpha (8–13 Hz), beta (13–30 Hz), and the sub-bands: low beta (13–20 Hz) and high beta (20–30 Hz). All the EEG-related measurements were calculated from three regions of interests (ROIs): the central, parietal, and frontal areas, since they are important cortical regions involved in pain perception^75^. Electrodes representing these regions were selected and averaged (the electrode placement is presented in the Supplementary Material S2).

### Statistical analysis

We used descriptive statistics to report baseline characteristics. Continuous data were expressed as mean and standard deviation (SD) or as median and interquartile ranges dependent on their distribution. Dichotomous and categorical data were described in frequency and respective percentages. Histogram and Shapiro–Wilk test assessed data distribution for normality. Values greater than 3 SDs away from the mean scores of the dependent or independent variables were labeled as outliers. After determining that data had a sufficiently normal distribution, we conducted exploratory multivariate linear regression models to identify relationships between resting EEG spectral power values (dependent variables) and clinical, QST, and TMS variables (independent variables). The models were conducted independently by frequency bands (delta, theta, alpha, beta, low-beta, and high-beta) and by region (frontal, central, and parietal). First, to select the best explanatory covariates, univariate linear models were created with each independent variable to detect significant covariates for an alpha level of 0.2. Variables that were not significant at the univariate models were eliminated. As a second step, a model was created with all variables that were significant (p < 0.2) in the univariate models. Thirdly, the regression coefficients were then checked for significance and those with a p-value > 0.05 were excluded from the model leaving only the significant variables (p < 0.05). Finally, to select our final multivariate modes, we search for confounders using a multicriteria approach: (1) based on previous literature supporting physiological plausibility, (2) considering changes of β coefficients more than 10%, and (3) using the Akaike’s information criteria to select the variables that would result in the best fit^76,77^. We also tested the interaction of demographic and clinical variables with the main predictors’ variables which was included in the final models if significant. Age and gender were explored as biological variables that could potentially confound all final models. Once the final model was determined, we added these variables as covariates, and if not significant, they were excluded from the models.

The assumption of linearity was assessed by visually comparing the scatterplot of each independent variable and a superimposed regression line. The assumption of homoscedasticity was checked by visual inspection of the scatterplot of the standardized predicted values and standardized residuals^78^. Residuals were tested for normality using histograms and the Shapiro–Wilk normality test^79^. Durbin Watson estimates and Cook’s distances were used for analysis of regression diagnostics such as multicollinearity and influential cases.

We used Stata Statistical Software 15 (Stata Corp LLC) for the statistical analyses. Because this was an exploratory study and to minimize the risk of type II errors, no correction for multiple comparisons was done.

## Supplementary Information

Supplementary Information 1.

Supplementary Information 2.
