# Supplementary material for: EEG theta and beta bands as brain oscillations for different knee osteoarthritis phenotypes according to disease severity
Source: Sci Rep. 2022 Jan 27;12:1480. doi: 10.1038/s41598-022-04957-x (PMC8795380; doi:10.1038/s41598-022-04957-x)
Supplement: Supplementary file 1 — Supplementary Information 1. [file 41598_2022_4957_MOESM1_ESM.docx]

**EEG theta and beta bands as brain oscillations for different knee osteoarthritis phenotypes according to disease severity**

Marcel Simis^1^, Marta Imamura^1^, Kevin Pacheco-Barrios^2,3^, Anna Marduy^2^, Paulo Sampaio de Melo^2^, Augusto J. Mendes^2,4^, Paulo E.P. Teixeira^2^, Linamara Battistella^1^, Felipe Fregni^2^

**Supplementary material S1**

**Functional Clinical Assessment Description and Application**

*Adapted from our protocol under review – Deficit of inhibition as a marker of neuroplasticity - DEFINE study - in rehabilitation: a longitudinal cohort study protocol - Sims et al.

Montreal Cognitive Assessment (MOCA): Screens the patient’s dominance over their cognitive function’s executive function, visuo-spatial ability, memory, attention, concentration, occupational memory, language, and temporal and spatial orientation. The scale has a maximum score of 30 points and an application time of approximately 10 minutes. In this scales lower scores mean more cognitive impairment.^1^

Pain Catastrophizing Scale: Made up of nine items staggered on a Likert scale, varying from 0-5 points related to the words “almost never” and “almost always” on the extremities. The total score is the sum of the items divided by the number of answered items, with the minimum achievable score being 0 and the maximum 5. Higher scores indicate the presence of catastrophizing thoughts.^2,3^

Visual Analogue Scale (VAS) for Pain: Made up of a 10 cm straight line on a piece of paper. Written on one of its ends is the phrase “no pain” and on the other “maximum pain”. Each subject was asked to mark their discomfort level on the VAS line. The closer the mark is to the scale’s origin (zero centimeters), the lower the patient’s pain level. On the other hand, the closer the mark is to the scale’s end (ten centimeters), the higher the patient’s pain level. Instructions for the patient was “Identify the amount of pain experienced in the last 48h and make a mark perpendicular to the ‘no pain’ – ‘maximum pain’ line”.^4^

6-minute and 10 meters gait test:^5^

10-meter gait test: aims to assess a patient’s short-distance walk speed. It is recommended that the subject walks 14 meters so that the 2 initial and final meters be disregarded. The subject walked at their normal speed.

6-minute gait test: evaluates the maximum distance a subject can walk on a plane, rigid surface in six minutes through a 30-meter track. It is recommended that this be a 30-meter walk, lapped every 3 meters in which turning points are set with a cone.

Timed Up and Go (TUG): This test evaluates an individual’s mobility level, measuring the amount of time it takes for the subject to stand up from a chair without using their arms, walk a 3-meter distance, turn 180o and get back to sit on the chair.^6^

Berg Balance Scale: this scale is made up of 14 tasks that assess static and dynamic balance, through tasks such as reaching, turning, transferring, standing, and standing up. The score for each item ranges from 0-4, determined by the ability to perform the task, and the maximum score is 56 points. In this scale, higher scores mean a better balance.^7^

Epworth sleepiness scale: Evaluates the degree of daytime sleepiness. It is a self-applied questionnaire that evaluates the probability of falling asleep in 8 everyday situations.^8^

Hamilton Depression Rating Scale (HAM-D): The instrument investigates how the patient has been feeling in the last seven days, including the day of application; it consists of 17 items, which can be scored on a Likert scale ranging from 0 to 2 or 0 to 4, depending on the intensity of the symptom. The total number of points varies between 0 and 52 points. To verify the presence of depression, the scores must add up to at least 8 points in the original version.^9^

Hospital Anxiety and Depression Scale (HADS): A 14-item scale that quantifies and qualifies symptoms of anxiety and depression. The scale contains 14 multiple-choice questions. It consists of two subscales, one for anxiety and another for depression, with seven items each. The global score in each subscale ranges from 0 to 21. It is intended to detect mild degrees of affective disorders in non-psychiatric environments. The patient is asked to respond based on how he felt during the last week.^10^

Western Ontario and McMaster Universities Osteoarthritis Index (WOMAC): Scale used to measure pain, stiffness, and physical function in patients with knee and hip osteoarthritis. The questionnaire consists of 24 items, divided into 3 subscales: pain, addressing 5 items, stiffness, 2 items, and function, 17 items. This scale usually is rated from 0 to 4, with lower scores indicating lower levels of symptoms or physical disability. The subscales are summated in a total of 20 for pain, 8 for stiffness, and 68 for physical function. There is also a global score that is calculated summating all the three domains.^11^

Kellgren-Lawrence Radiographic Classification of OA: it is a method of classifying the severity of knee osteoarthritis (OA), which uses five degrees: 0 (without osteoarthritis) to 4 (large osteophyte, marked narrowing of the joint space, severe sclerosis, and definite deformity of bony extremities).^12^

36-item short form (SF-36): The SF-36 includes a multi-item scale that access eight health concepts: 1) physical functioning (10 items and 21 levels); 2) Role limitations due physical problems (4 items and 5 levels); 3) social functioning (2 items and levels); 4) bodily pain (2 items and 11 levels); 5) general mental health (5 items and 26 levels); 6) role limitations due emotional problems (3 items and 4 levels); 7) vitality (4 items and 21 levels); and 8) general health perceptions (5 items and 21 levels). In these scales, lower scores mean a lower health status, while higher scores mean a higher health status.^13^

**REFERENCES**

1 Freitas, S., Simões, M. R., Alves, L. & Santana, I. Montreal Cognitive Assessment (MoCA): normative study for the Portuguese population. *J Clin Exp Neuropsychol* **33**, 989-996, doi:10.1080/13803395.2011.589374 (2011).

2 Sullivan, M. J. L., Bishop, S. R. & Pivik, J. The Pain Catastrophizing Scale: Development and validation. *Psychological Assessment* **7**, 524-532, doi:10.1037/1040-3590.7.4.524 (1995).

3 Sardá Junior, J. *et al.* Validation of the Pain-Related Catastrophizing Thoughts Scale. *Acta Fisiátrica* **15**, doi:10.5935/0104-7795.20080001 (2008).

4 Williamson, A. & Hoggart, B. Pain: a review of three commonly used pain rating scales. *J Clin Nurs* **14**, 798-804, doi:10.1111/j.1365-2702.2005.01121.x (2005).

5 Steele, B. Timed walking tests of exercise capacity in chronic cardiopulmonary illness. *J Cardiopulm Rehabil* **16**, 25-33, doi:10.1097/00008483-199601000-00003 (1996).

6 Podsiadlo, D. & Richardson, S. The timed "Up & Go": a test of basic functional mobility for frail elderly persons. *J Am Geriatr Soc* **39**, 142-148, doi:10.1111/j.1532-5415.1991.tb01616.x (1991).

7 Stevenson, T. J. Detecting change in patients with stroke using the Berg Balance Scale. *Aust J Physiother* **47**, 29-38, doi:10.1016/s0004-9514(14)60296-8 (2001).

8 Johns, M. W. Reliability and factor analysis of the Epworth Sleepiness Scale. *Sleep* **15**, 376-381, doi:10.1093/sleep/15.4.376 (1992).

9 Freire, M. Á. *et al.* Escala Hamilton: estudo das características psicométricas em uma amostra do sul do Brasil. *Jornal Brasileiro de Psiquiatria* **63**, 281-289, doi:10.1590/0047-2085000000036 (2014).

10 Botega, N. J., Bio, M. R., Zomignani, M. A., Garcia, C., Jr. & Pereira, W. A. [Mood disorders among inpatients in ambulatory and validation of the anxiety and depression scale HAD]. *Rev Saude Publica* **29**, 355-363, doi:10.1590/s0034-89101995000500004 (1995).

11 McConnell, S., Kolopack, P. & Davis, A. M. The Western Ontario and McMaster Universities Osteoarthritis Index (WOMAC): a review of its utility and measurement properties. *Arthritis & Rheumatism* **45**, 453-461, doi:10.1002/1529-0131(200110)45:5<453::aid-art365>3.0.co;2-w (2001).

12 Kellgren, J. H. & Lawrence, J. S. Radiological assessment of osteo-arthrosis. *Ann Rheum Dis* **16**, 494-502, doi:10.1136/ard.16.4.494 (1957).

13 Ware, J. E., Jr. & Sherbourne, C. D. The MOS 36-item short-form health survey (SF-36). I. Conceptual framework and item selection. *Med Care* **30**, 473-483 (1992).
