# Supplementary material for: EEG theta and beta bands as brain oscillations for different knee osteoarthritis phenotypes according to disease severity
Source: Sci Rep. 2022 Jan 27;12:1480. doi: 10.1038/s41598-022-04957-x (PMC8795380; doi:10.1038/s41598-022-04957-x)
Supplement: Supplementary file 2 — Supplementary Information 2. [file 41598_2022_4957_MOESM2_ESM.docx]

**EEG theta and beta bands as brain oscillations for different knee osteoarthritis phenotypes according to disease severity**

Marcel Simis^1^, Marta Imamura^1^, Kevin Pacheco-Barrios^2,3^, Anna Marduy^2^, Paulo Sampaio de Melo^2^, Augusto J. Mendes^2,4^, Paulo E.P. Teixeira^2^, Linamara Battistella^1^, Felipe Fregni^2^

**Supplementary material S2**


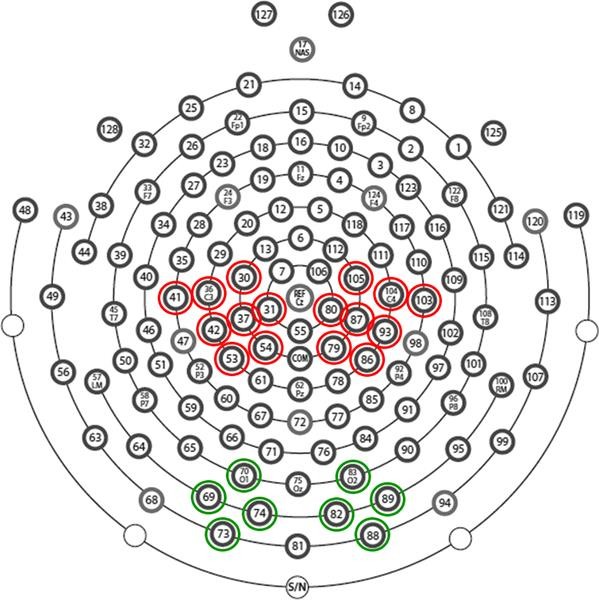

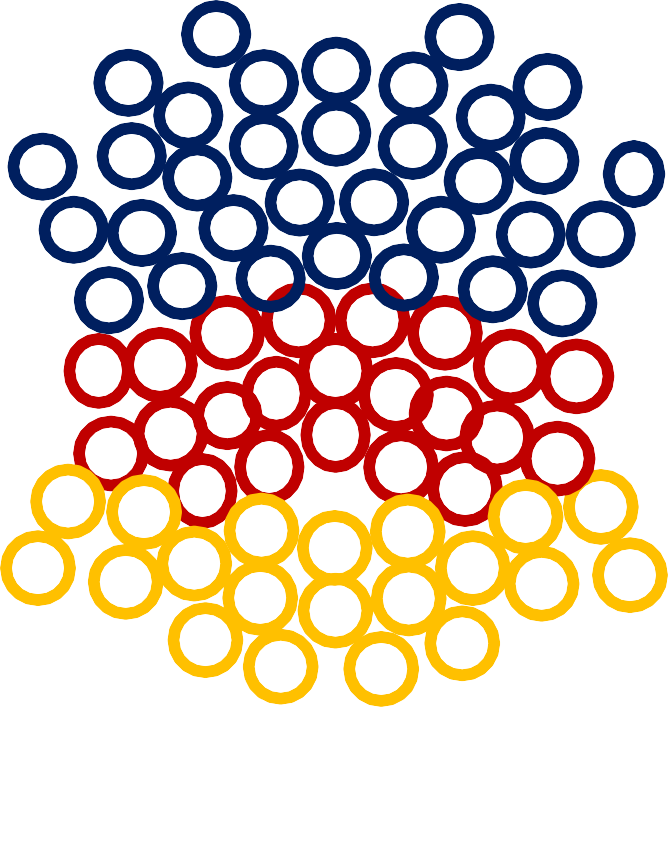


Frontal

Central

Parietal
